# Minute ventilation at different compression to ventilation ratios, different ventilation rates, and continuous chest compressions with asynchronous ventilation in a newborn manikin

**DOI:** 10.1186/1757-7241-20-73

**Published:** 2012-10-17

**Authors:** Anne L Solevåg, Jorunn Marie Madland, Espen Gjærum, Britt Nakstad

**Affiliations:** 1 The Department of Children and Adolescents, Akershus University Hospital, 1478, Lørenskog, Norway; 2 University of Oslo, 0316, Oslo, Norway

**Keywords:** Newborn, Resuscitation, Positive-pressure respiration, Heart massage, Pulmonary ventilation, Manikin

## Abstract

**Background:**

In newborn resuscitation the recommended rate of chest compressions should be 90 per minute and 30 ventilations should be delivered each minute, aiming at achieving a total of 120 events per minute. However, this recommendation is based on physiological plausibility and consensus rather than scientific evidence. With focus on minute ventilation (Mv), we aimed to compare today’s standard to alternative chest compression to ventilation (C:V) ratios and different ventilation rates, as well as to continuous chest compressions with asynchronous ventilation.

**Methods:**

Two investigators performed cardiopulmonary resuscitation on a newborn manikin with a T-piece resuscitator and manual chest compressions. The C:V ratios 3:1, 9:3 and 15:2, as well as continuous chest compressions with asynchronous ventilation (120 compressions and 40 ventilations per minute) were performed in a randomised fashion in series of 10 × 2 minutes. In addition, ventilation only was performed at three different rates (40, 60 and 120 ventilations per minute, respectively). A respiratory function monitor measured inspiration time, tidal volume and ventilation rate. Mv was calculated for the different interventions and the Mann–Whitney test was used for comparisons between groups.

**Results:**

Median Mv per kg in ml (interquartile range) was significantly lower at the C:V ratios of 9:3 (140 (134–144)) and 15:2 (77 (74–83)) as compared to 3:1 (191(183–199)). With ventilation only, there was a correlation between ventilation rate and Mv despite a negative correlation between ventilation rate and tidal volumes. Continuous chest compressions with asynchronous ventilation gave higher Mv as compared to coordinated compressions and ventilations at a C:V ratio of 3:1.

**Conclusions:**

In this study, higher C:V ratios than 3:1 compromised ventilation dynamics in a newborn manikin. However, higher ventilation rates, as well as continuous chest compressions with asynchronous ventilation gave higher Mv than coordinated compressions and ventilations with 90 compressions and 30 ventilations per minute.

## Background

Five to ten percent of newborns need assistance to establish breathing at birth [1-5]. Even though scientific studies have not addressed the optimal ventilation rate when positive pressure ventilation (PPV) is required in the delivery room, The International Liaison Committee on Resuscitation (ILCOR) guidelines state that 40 to 60 ventilations per minute might be appropriate [6]. A commonly used tidal volume (V_T_) in assisted ventilation of newborns is 4–8 ml/kg [7]. There is insufficient evidence to recommend an optimal inflation time.

If 30 seconds of effective PPV do not lead to a rise in heart rate above 60 beats per minute, chest compressions should be initiated with a compression:ventilation (C:V) ratio of 3:1. The rate of chest compression should be 90 per minute and 30 inflations should be delivered each minute during cardiopulmonary resuscitation (CPR), aiming at achieving a total of 120 events per minute [8]. However, this recommendation is based on physiological plausibility and consensus, rather than scientific evidence.

We aimed to investigate different C:V ratios, ventilation rates, as well as continuous chest compressions with asynchronous ventilation with regards to delivered V_T_ and minute ventilation (Mv) by using a newborn manikin, a T-piece resuscitator and a respiratory function monitor.

Our research question was whether alternative C:V ratios, ventilation rates or continuous chest compressions with asynchronous ventilation would compromise ventilation as measured by reduced Mv in a newborn manikin.

## Methods

### Technical equipment

We used the SimNewB™ (Lærdal Medical AS, Stavanger, Norway), simulating a 3.5 kg newborn baby. The SimNewB™ is an advanced simulator with a range of options with regards to airway characteristics, including three settings for airway restriction and airway compliance. We used the simulator with the default settings from the manufacturer with fully open airways and no noticeable restrictions during ventilation. In order to deliver PPV, the Neopuff™ T-piece resuscitator (Fisher & Paykel Healthcare, Auckland, New Zealand) was used; medical air being used to generate a flow of 8 l/min. In a pilot experiment we found that a positive end-expiratory pressure (PEEP) of 8 cm H_2_O and a peak inspiratory pressure (PIP) of 30 cm H_2_O were appropriate in order to obtain tidal volumes of 4–8 ml/kg in this model. The Laerdal Silicone Infant Mask No 0/1 was the facemask in use. A SensorMedics Vmax 26 (Eco Medics AG, Switzerland) was used to measure V_T_, ventilation rates, inspiration times (T_I_) and expiration times (T_E_). In order to be able to deliver chest compressions and ventilations at the assigned rate, we used a web-based metronome (webmetronome.com).

The SensorMedics Vmax 26 has a dead volume of less than 2 ml in the standard setup for infant flow monitoring. We added a connector between the Neopuff™ and this device, adding less than 0,5 ml of dead space volume. The Vmax was set up for standard infant flow measurements (i.e. default settings) as indicated by the manufacturer, except from a volume deviation of 100%. Analogue signals in the Vmax are being digitised and analysed using the Spiroware® recording software with the tidal breathing flow volume loop (TBFVL)-INF program. Filter size 1 with dead space reducer (Eco Medics, DSR Set 1 (small)) was used. With this instrument, inspiratory volume (V_TI_) and expiratory volume (V_TE_) are automatically calculated by integrating the flow signals and V_T_ is calculated as the mean of V_TI_ and V_TE_. The software continuously displays gas flow and V_T_ waves. In addition, it measures and displays numerical values for V_TI_, V_TE_, T_I_, T_E_ and respiratory rate.

### Optimizing ventilation and chest compressions

The main study was preceded by a pilot study where we tested different ways of delivering PPV to the SimNewB™ in order to minimize face/mask leak.

1) Nasal and oral endotracheal tube

a) Self-inflating bag

b) Neopuff™

2) Bag-mask ventilation directly on the manikin’s face, covering the nose and mouth with the mask.

With the mask being placed over the nose and the mouth of the manikin, there was a significant leak between the rubber ”skin” of the manikin and the face. To overcome this problem, the ”skin” was removed, and the mask was placed directly onto the face of the manikin.

The investigators practised ventilation of this model until they managed to ventilate the manikin without a significant mask leak (as measured by V_TI_ ≈V_TE_). The mask was attached to the flow sensor of the SensorMedics Vmax 26, and further onto the Neopuff™.

Chest compressions were performed using the two-thumb-encircling hands technique. In the pilot study, the investigators practised their technique for performing chest compressions with correct finger placement on the manikin chest and depth of compressions being approximately one third of the anterior-posterior diameter of the chest.

### Experimental protocol

In this study, we chose to investigate the ratio of 9:3 chest compressions to ventilations because in theory it will, in the clinical setting, produce a higher coronary perfusion pressure due to more compressions in a series, and at the same time maintain ventilation as compared to 3:1. A C:V ratio of 15:2 was chosen because this is the currently recommended ratio in all patients when advanced CPR is performed, except in newborns.

Two investigators, JMM and EG performed CPR on the SimNewB™ with the C:V ratios 3:1, 9:3 and 15:2, as well as continuous chest compressions with asynchronous ventilation (120 compressions and 40 ventilations per minute). JMM and EG were undergraduate medical students and had no practical experience in neonatal resuscitation. As previously mentioned, they practiced ventilation and chest compressions in this model to an extent that made them capable of delivering consistent CPR without signs of fatigue as measured by stable ventilation parameters during the 2-minute series of CPR.

A commonly used duration of CPR cycles in this type of research is 90 seconds or 2 minutes [9,10]. We chose to perform each intervention in series of 10 × 2 minutes. The different interventions were randomized and a metronome was used to guide CPR at a rate of 120 events per minute as recommended by international guidelines for newborn resuscitation [6].

In addition, ventilation only was performed at three different rates (40, 60 and 120 ventilations per minute, respectively).

The alternative interventions were compared to the currently recommended ratio of 3:1 chest compressions to ventilations with a total rate of 120 events per minute

The different interventions are presented in Table 1.

**Table 1:** Ventilation characteristics at different compression:ventilation ratios, continuous chest compressions with asynchronous ventilation; and ventilation rates

**Intervention**	**Number of ventilations/min**	p-value*	**V**_**T**_**(ml) Absolute**	p-value*	**V**_**T**_**(ml) Per kg**	p-value*	**Mv (ml)**	p-value*	**Mv (ml) Per kg**	p-value*	**T**_**I**_**(s)**	p-value*
Continuous chest compressions with asynchronous ventilation	39 (39–39)	<0.001	19.7 (18.8-21.1)	0.002	5.6 (5.4-6.0)	0.002	777 (735–836)	<0.001	221 (210–239)	<0.001	0.52 (0.49-0.58)	0.427
C:V = 3:1 120 events per minute	30 (29–30)		22.3 (21.9-23.8)		6.4 (6.3-6.8)		668 (642–697)		191 (183–199)		0.50 (0.48-0.55)	
C:V 9:3 120 events per minute	28 (28–30)	0.057	17.1 (16.5-17.1)	<0.001	4.9 (4.8-5.1)	<0.001	490 (468–506)	<0.001	140 (134–144)	0.002	0.33 (0.33-0.34)	<0.001
C:V 15:2 120 events per minute	14 (13–15)	<0.001	19.1 (19.0-19.9)	<0.001	5.5 (5.4-5.7)	<0.001	268 (261–289)	<0.001	77 (74–83)	<0.001	0.33 (0.32-0.34)	0.001
Ventilations only 40 per minute	40 (40–40)	<0.001	19.5 (18.8-19.9)	<0.001	5.6 (5.4-5.7)	<0.001	760 (734–775)	<0.001	217 (210–221)	<0.001	0.59 (0.57-0.71)	0.003
Ventilations only 60 per minute	58 (58–59)	<0.001	17.4 (16.8-17.8)	<0.001	5.0(4.8-5.1)	<0.001	1013 (973–1028)	<0.001	289 (278–294)	<0.001	0.56 (0.53-0.60)	0.049
Ventilations only 120 per minute	118 (117–118)	<0.001	13.1 (13.1-13.6)	<0.001	3.8 (3.7-3.9)	<0.001	1544 (1525–1595)	<0.001	441 (436–456)	<0.001	0.24 (0.24-0.25)	<0.001

Values are shown as median (IQR) *compared to a chest compression to ventilation ratio of 3:1.

C:V = chest compressions to ventilations, V_T_ = tidal volume, Mv = minute ventilation, T_I_ = inspiratory time.

### Statistical calculations

Statistical analyses were performed using SPSS 15.0 for Windows (SPSS Inc., Chicago, Ill., USA).

Based on the pilot experiment the study was powered to detect a difference in 0.25 ml V_T_ (per kg) between the groups with a type I error rate of 5% and a power of 80%. The calculated number of experiments (series) needed for each intervention was then 6. Because performing more experiments did not pose ethical challenges as in clinical studies, we decided on repeating each intervention 10 times.

As ventilatory parameters were not normally distributed, we report descriptive statistics as median and interquartile range (IQR). The Mann–Whitney test was used for comparisons between groups.

## Results

### General

As the experiments were metronome-guided, we managed to achieve a number of ventilations at the different rates and ratios close to target (Table 1). At the C:V ratio of 3:1 and a total of 120 events per minute, median number of ventilations per min was 30, whereas at the ratio of 9:3, we achieved a median of 28 ventilations per minute. Likewise, in the case of ventilations only, the number of ventilations achieved approximated the target (Table 1). In addition, V_T_ was within the recommended range of 4–8 ml/kg in all interventions except ventilations only at a rate of 120 per minute (Table 1). There was a highly significant correlation between T_I_ and V_T_ in all groups (p<0.001).

### Different compression to ventilation ratios

The C:V ratio of 3:1 gave both higher T_I_ and V_T_ than the ratio of 9:3, giving a significantly higher minute volume (Mv) in ml per kg with 3:1 compressions to ventilations: 191(183–199) (median (IQR)) than in the 9:3 group: 140 (134–144) (p<0.001). The ventilation rate (median (IQR)) at a C:V ratio of 15:2 (14 (13–15) per minute) was significantly lower than at a ratio of 3:1 (30 (29–30) per minute) giving a significantly lower Mv in ml per kg with 15:2 compressions to ventilations (77 (74–83)) (p<0.001) (Table 1).

### Ventilation only at different rates

Even though T_I_ and V_T_ were inversely correlated to the ventilation rate, the higher rates (i.e. 60 and 120 per minutes) gave higher Mv per kg than a ventilation rate of 40 per minute (Table 1), demonstrating a correlation between Mv and ventilation rate.

### Continuous chest compressions with asynchronous ventilation

Continuous chest compressions with asynchronous ventilation gave lower V_T_ than coordinated compressions and ventilations at a ratio of 3:1 (p=0.002). However, due to a higher number of ventilations per minute, a higher Mv per kg than with the currently recommended C:V ratio of 3:1was achieved (p<0.001) (Table 1).

## Discussion

In this study we demonstrated that Mv was significantly reduced at alternative C:V ratios such as 9:3 and 15:2 compared to the standard ratio of 3:1. At the 9:3 ratio this was caused by a shorter T_I_ leading to lower V_T_; whereas at the 15:2 ratio, reduced Mv was mainly caused by a lower ventilation rate. With ventilation only, ventilation rate was correlated to Mv. Interestingly, continuous chest compressions with asynchronous ventilation gave higher Mv than the currently recommended C:V ratio of 3:1 due to a higher rate of ventilations.

### C:V ratios

In a recent manikin study by Hemway et al., the C:V ratio of 3:1 was found to generate more ventilations per 2-minute CPR cycle than the C:V ratios of 5:1 and 15:2. However, that study did not evaluate the effectiveness of ventilations at the different ratios [10]. Assuming that ventilation is crucial in newborn resuscitation, the 3:1 C:V ratio seems to be the most favourable based on both the present study and the study by Hemway et al.

### Ventilation rates

Important clinical studies investigating different assisted ventilation rates in the newborn were performed in the 1980’s [11,12]. Field et al. showed that mechanical ventilation at a rate of 100 per minute resulted in better oxygenation than lower rates [11]. However, as the authors proposed that the advantageous effect of increasing the ventilator rate was due to a suppression of spontaneous breathing, these results cannot be readily transferred to resuscitation in the delivery room.

### Continuous chest compressions with asynchronous ventilation

In children (except for the newly born) and adults, continuous chest compressions with asynchronous ventilation are recommended once an advanced airway is in place [13,14]. With regards to newborns, two studies of infant pigs have compared uncoordinated compressions and ventilations to coordinated chest compressions and ventilations at a ratio of 5:1 [15,16]. Berkowitz et al. concluded that the uncoordinated approach might be advantageous for myocardial blood flow during brief periods of cardiac arrest [16]. However, methodological concerns related to these studies, as well as a lack of human data make guideline development difficult, especially with regards to the question of the safety and effectiveness of continuous chest compressions with asynchronous ventilation in the newborn.

Hence, continuous chest compressions with asynchronous ventilation have to be studied in different models before change in practice can be recommended. Resuscitator preferences and exhaustion at the different ratios and rates, as well as at continuous compressions with asynchronous ventilation can influence outcome in the clinical setting. However, our results do indicate that continuous compressions with asynchronous ventilation may be safe with regards to ventilation also in the newborn.

### The manikin model

Manikins have been used extensively in the study of mechanical aspects of CPR [17,18]. Various technical features of the manikins, as well as the structure and size of the manikins make different studies more or less comparable. In neonatal CPR a commonly used manikin is the Laerdal Heart Code BLS that is suitable for recording chest compression dynamics [10,19]. However, as the Heart Code BLS manikin equals a 6 kg infant, we found it more appropriate to use the SimNewB™ that according to Laerdal is more similar to a 3.5 kg infant in terms of size, compliance of the chest and airways, as well as the relationship between the airways and the thoracic wall. However, as the Laerdal manikins are primarily developed for educational purposes (the SimNewB™ does not record chest compression mechanics precise enough for this type of research), the results of scientific studies performed with these models should be interpreted with a certain degree of caution.

Still, we argue that manikin studies can provide knowledge about ventilation dynamics at alternative C:V ratios, ventilation rates and continuous chest compressions with asynchronous ventilation. In this study we measured the effect of different C:V ratios and ventilation rates on ventilation volumes, demonstrating plausible effects: Increasing ventilation rates makes each ventilation shorter which in turn reduces V_T_; and increasing the number of ventilations increases Mv. But, despite the predictability of the results, these hypotheses have never been tested in scientific studies prior to this.

Even though our manikin was not intubated, continuous chest compressions with asynchronous ventilation did not interfere with ventilation as measured by a higher Mv with this approach as compared to today’s standard of 3:1 chest compressions to ventilations. However, as our experiments were optimised to achieve standardised ventilations with minimal face/mask leak, the situation is not entirely comparable to CPR in the delivery room where the amount of mask leak might be different at different ventilation/compression interventions.

As the results of the different interventions were strikingly consistent with low variability (narrow IQRs) for Mv within each intervention (Table 1), we find that our model is robust and reliable. Also, as we almost uniformly achieved V_T_ within the recommended range of 4–8 ml/kg, this adds to the notion that the model we used is suitable for investigating ventilatory dynamics in the newborn. However, as opposed to a newborn undergoing pulmonary transition with changes in the amounts of liquid in the alveoli during resuscitation, lung compliance was high (no airway resistance) and fixed in our manikin model.

However, as the aim of the study was to measure differences between alternative measures, we believe that our results add to the knowledge about how ventilation is influenced by different C:V ratios and ventilation rates.

Higher ventilation rates improved Mv in this study. However, it is not known how easily these rates can be achieved in a real life setting and how this may influence clinical outcome.

Importantly, this study only investigated one aspect of CPR, delivery of Vt and Mv. In the clinical setting, cardiac output is influenced by chest compression. As oxygenation depends both on ventilation and cardiac output, the entire truth about the influence of different C:V ratios cannot be found through manikin research.

### The investigators

As opposed to the participants in the study by Hemway et al. who mostly were health care workers in neonatology [10], the subjects performing CPR in this study were probably not biased toward any intervention.

These manikin studies are valuable since the questions being addressed are difficult to examine in clinical studies, as significant ethical issues concerning resuscitation research in the newborn would have to be taken into account. However, further studies investigating the optimal C:V ratio and ventilation rates; as well as continuous chest compressions with asynchronous ventilation in newborn resuscitation should be undertaken.

## Conclusions

In a neonatal manikin, higher C:V ratios than the currently recommended 3:1 did compromise ventilation dynamics as measured by reduced Mv. However, continuous chest compressions with asynchronous ventilation increased Mv, which may be desirable in asphyxiated infants.

Alternatives to today’s CPR standards may be easier to perform in real life and this can affect outcome in the clinical setting. Also, the quality of chest compressions at different C:V ratios is thought to influence outcome in newborn CPR. We did not measure chest compression characteristics in this study. However, this together with rescuer preferences and pedagogical concerns should be investigated further as they may possibly influence guideline development.

## Abbreviations

PPV: Positive pressure ventilation; ILCOR: The International Liaison Committee on Resuscitation; V_T_: Tidal volume; C:V: Compression:ventilation; CPR: Cardiopulmonary resuscitation; Mv: Minute ventilation; PEEP: Positive end-expiratory pressure; PIP: Peak inspiratory pressure; T_I_: Inspiratory time; T_E_: Expiratory time; V_TI_: Inspiratory volume; V_TE_: Expiratory volume.

## Competing interests

The authors declare that they have no competing interests. The study was not funded by external sources.

## Authors’ contributions

ALS and BN conceived of the idea for the study and drafted the research protocol. JMM and EG refined the protocol and carried out the experiments in close collaboration with ALS and BN. ALS performed the statistical analysis and drafted the manuscript. All authors read and approved the final manuscript.
